# Home-Based Care for People with Alzheimer’s Disease and Related Dementias (ADRD) during COVID-19 Pandemic: From Challenges to Solutions

**DOI:** 10.3390/ijerph17249303

**Published:** 2020-12-12

**Authors:** Atiqur sm-Rahman, Chih Hung Lo, Azra Ramic, Yasmin Jahan

**Affiliations:** 1 Department of Culture and Society, Division Ageing and Social Change, Linkoping University, 601 74 Norrkoping, Sweden; 2 Department of Neurology, Brigham and Women’s Hospital, Harvard Medical School, Boston, MA 02115, USA; chihhunglo88@gmail.com; 3 Stroke Unit, Clinical Medicine, Vrinnevi Hospital, Norrköping-Region Östergötland, 603 79 Norrköping, Sweden; ramic_azra@hotmail.com; 4 Graduate School of Biomedical and Health Sciences, Hiroshima University, Hiroshima 739-8527, Japan; dr.yasminjahan@gmail.com

**Keywords:** dementia, ADRD, COVID-19, pandemic, home-based care, policy

## Abstract

There has been supporting evidence that older adults with underlying health conditions form the majority of the fatal cases in the current novel coronavirus disease (COVID-19) pandemic. While the impact of COVID-19 is affecting the general public, it is clear that these distressful experiences will be magnified in older adults, particularly people living with Alzheimer’s disease and related dementia (ADRD), making them the most vulnerable group during this time. People with differing degrees of ADRD are especially susceptible to the virus, not only because of their difficulties in assessing the threat or remembering the safety measures, but also because of the likelihood to be subject to other risk factors, such as lack of proper care and psychological issues. Therefore, in this article, we will discuss the challenges related to home-based care for people with ADRD during a pandemic and propose a formulation of systematic solutions to address these challenges and to alleviate the social and economic impact resulting from the crisis.

## 1. Background

The world is currently grappling with a rapid evolving pandemic of the novel coronavirus disease (COVID-19). Older adults aged 65 and above have been defined as a higher risk group of the virus in scientific publications as well as in media coverage [1,2]. This group of people, therefore, has become the subject of specific national social distancing and ‘cocooning’ measures [3]. These measures and implications are difficult to be handled by older adults, in particular, individuals with Alzheimer’s disease and related dementias (ADRD), due to implications from disease symptoms and the need for additional care [4]. It is unclear from existing discussions how the COVID-19 pandemic is affecting people with ADRD as a whole, even though they respond to the crisis differently based on their stages in disease progression [5,6]. There is a lack of study showing a direct association between the suspected, infected, or fatal cases of COVID-19 and people with ADRD thus far. More importantly, how exactly people with ADRD are responding to the pandemic situation remains unknown.

A further complication to this is that there is no specific policy or planning from any country on how to deal with people with ADRD who are suffering from COVID-19 [7,8]. This is an alarming oversight which is not explicitly addressed by the World Health Organization [9] and appeared as a cornerstone that has received negligible attention in the new policy announced by the United Nations [10]. Hence, without delineating an accurate number of COVID-19 positive people with ADRD from the general reported cases [11], there is a high possibility of overlooking the actual home-based care needs for the people with ADRD and this could affect the timely development of suitable policies for this group of people [12].

## 2. COVID-19 and People with ADRD

ADRD, resulting from neurodegeneration, becomes more common with age [13,14,15,16] and predominantly affects older adults, particularly from middle age (40s, 50s and early 60s) onwards [5]. Globally, there is an estimate of 50 million people living with dementias [17] and 1 in 14 people over the age of 65 and 1 in 6 people over the age of 80 are diagnosed with ADRD [4]. Many of them may not be in regular contact with the health and social care system and may not even have sufficient information about COVID-19. According to the Centers for Disease Control and Prevention (CDC), older adults aged 65 and above have been uniformly defined as a ‘higher risk group’ with regards to the virus [18,19], regardless of their associated health comorbidities. People with ADRD are described as not only susceptible to developing severe COVID-19 symptoms but are also less responsive to treatments due to other existing health conditions [19,20,21,22].

Prominent clinical features of COVID-19, such as hypoxia or respiratory distress, cause delirium [23,24] and sudden cardiac arrest, which could create complications for COVID-19 treatment for people with ADRD [25]. In particular, the higher risk of delirium with COVID-19 could be misdiagnosed as a form of dementia and therefore affect immediate treatment [26,27]. Furthermore, memory problems at late-stage ADRD, which, for example, make safety instructions difficult to remember [28,29] and also make clearly understanding the messages of cocooning and self-isolation difficult [30,31], are deteriorating the capabilities of this group of people to combat the pandemic. The pathophysiological processes of ADRD do not increase the risk of COVID-19; however, they may be subject to other risk factors such as anxiety, trauma, loneliness, existential uncertainty, and social isolation [22].

The dynamic nature of COVID-19 corresponds with the severe acute respiratory syndrome (SARS-2003), which strongly highlights the urgent need to develop suitable policies for older adults and ADRD care. Retrospective studies, for instance, found that rates of suicidal tendencies among older adults aged 65 and above were higher during the SARS-2003 epidemic [32,33,34]. The study further identified predominant reasons explaining this exceptional increase of suicide deaths among older adults, which include anxiety in contracting the disease, fear of disconnection, stress over being a burden to their families, and social disengagement. The combination of these reasons was described as a “serious public health concern” [35] that must be addressed immediately to minimize the adverse impact of the pandemic on people with ADRD. A better understanding and appreciation of the impact of this rapidly evolving situation can help in the improvement of care for people with ADRD [36]. Besides, the documentation of measures taken by institutions (structural) and individuals (personal) to manage the effect of COVID-19 will facilitate future strategies on disease prevention for people with ADRD [37].

Evidence shows that more than 40% of older adults living in long-term facilities (home- and community- based care) are affected disproportionately by COVID-19 [38]. Currently available literature also demonstrates that most of the people with ADRD living with family members prefer home-based care to nursing home [39]. This choice depends heavily on family caregivers as indispensable partners in dementia home care [40]. Home dwelling people with ADRD are experiencing challenges related to inadequate preventive measures and essential health services during the COVID-19 surge [38]. While emergency planning on acute care for people with ADRD living in nursing home (e.g., long-term care) was taken into consideration at the beginning of COVID-19 and has been addressed over time [29,41], it should be noted with concern that, to-date, very few existing preventive measures have targeted people with ADRD living at home with a personal caregiver (e.g., family member) or living alone by themselves [5].

This article advocates for a formulation of solutions to address the challenges related to home-based care for people with moderate to severe ADRD under pandemic situations such as COVID-19. We offer suggestions in order to frame an action plan in the field of ADRD home-based care to determine and discuss what the deciding factors are, so that people with ADRD can be protected more effectively. The goal is to propose corresponding recommendations to mitigate the challenges related to pandemic situations in a timely manner, which can further influence the decisions of caregivers, health professionals, and policymakers.

## 3. Challenges in Home-Based Care for People with ADRD

People with ADRD utilize various resources from different settings depending on their severity of illness [42]. They are among the most vulnerable groups in society due to the deterioration of physical and cognitive functionalities that often leads toward a high dependency on others [43]. A study has argued that the current pandemic has heightened the morbidity and mortality among people with ADRD and put restrictions on the social support and the health care system on which they depend [44]. COVID-19 thus generates additional care needs from both home-based care (providing low-intensity care, with few trained staff) and nursing home care (providing high-intensity care with trained nursing and medical staff) services [44,45,46].

Despite the available and accessible resources in long-term care or nursing home facilities, about one-third of people with moderate to severe ADRD live with family and on their own [47]. The pandemic generally disrupted or delayed delivering services such as Meals on Wheels for them due to increased demand [48], workers’ illness [49], or required isolation due to exposure [50]. Moreover, people with ADRD living at home had to discontinue their purposeful socio-cultural activities such as social engagement, group exercise, music therapy, and pet therapy because of prolonged physical distancing [48,51]. To stay socially connected, people are turning to technology, which can also be difficult to adopt for individuals with ADRD due to cognitive impairment [44,52]. These needs are less visible but no less important.

Similarly, the primary caregivers of people with ADRD are facing additional difficulties while following the new rules of social distancing and cocooning. The pandemic has made some of them mentally and physically precarious [44,53]. They may become ill, isolated, or unavailable. In the sudden unavailability of caregivers, relatives or friends may have to be responsible for taking care of a person with ADRD, which is incompatible with physical distancing.

Preventive measures like social distancing, physical distancing, and the restriction of visitors at home and in nursing cares, as well as home quarantine adopted by several countries worldwide, have been particularly challenging for people with ADRD [54]. For instance, it is not easy for people with late-stage ADRD to understand instructions about social distancing (e.g., staying “2 meters or 6 feet apart” from others) and abide by the rules. The ‘forgetful’ characteristics might hinder them from remembering to wear face masks, wash their hands frequently, or take other recommended preventive measures [29]. The limited access to information poses additional difficulties to the group to change or reconfigure personal and social expectations. Simultaneously, it is important to pay attention to how the lack of these preventive measures could impact the dynamic interactions between people with ADRD and their caregivers, leading to both having a higher chance of infection [44]. Therefore, we need to continuously reinforce basic health care messages in order to prevent or reduce the harm of the COVID-19 pandemic for both groups.

**Figure 1:**
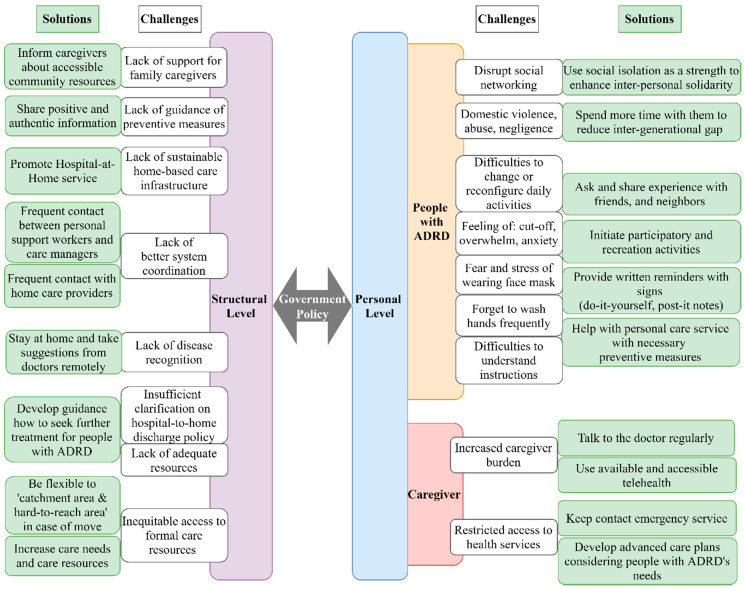
Challenges related to Alzheimer’s disease and related dementia (ADRD) in a pandemic and proposed solutions.

We classified the challenges and the possible solutions into two interconnected levels—structural level and personal level (Figure 1)—which may derive from the COVID-19 pandemic for people with ADRD. The structural challenges mostly illustrate policy and care infrastructure constraints, whereas the personal level explored the requirements for people with ADRD in a home-based care setting. Lessons learned from the previous epidemic crisis may enlighten our understanding of the best effective measures.

During a pandemic, the virus is not only threatening people with ADRD, but also their family members and friends, who are the main sources of home-based care in contexts where there is a lack of adequate health care systems. A significant proportion of people with ADRD has been suggested to have home-based care services with the argument of retaining social networks, individual independence, and a higher quality of life [55]. However, there are challenges related to home-based care, such as lack of support for family caregivers, lack of disease recognition, insufficient clarification in policies regarding hospital-to-home discharge, and poor system coordination [56]. Furthermore, the social distancing guidelines are almost impossible, particularly for those staying at home, due to limited resources for sustaining a home-based care infrastructure that allow the implementation of these measures.

Indeed, studies have suggested that the enforced isolation, quarantine, or lockdown of people with ADRD at home due to COVID-19 susceptibility can reduce transmission, minimize viral spread, and flatten the curve [35]. However, this will disrupt social networking and restrict access to health services for them, which are particularly important resources for people with ADRD. In addition, the increased dependency of people with ADRD on closed family members during a pandemic may result in them having higher risks of domestic violence, abuse, and negligence [57].

A lack of guidance for preventive measures, distressing news in the media, and rapidly changing information on viral infection can engender a greater risk of behavioral changes and confusion. Consequently, both people with ADRD and their family caregivers may face an unprecedented overwhelmed and anxious feeling, as well as an increasing risk of exposure to the virus in the context of the pandemic. The familial caregivers experience inequitable access to formal care resources that contributes to the strain while providing care to people with ADRD. A potential reason for the distress experienced by the caregivers could be due to the lack of awareness of the existing support services that they can utilize.

## 4. Proposed Solutions for Home-Based Care for People with ADRD

We propose an outline of ‘action plans’ to minimize home-based care challenges related to people with ADRD during the management of pandemic outbreaks (e.g., COVID-19). These solutions may generate ideas and thoughts among health professionals, care managers, and policy makers to develop and implement better home-based care interventions for the target population. Here, the action plan introduces a systematic process that includes fragmented ADRD care policies and appropriate ways of minimizing possible adverse consequences of viral spread or pandemic. That is, a multidisciplinary counseling team can develop an urgent guideline and offer self-help guidance, for example, in association with ADRD experts, psychologists, clinicians, social workers, occupational therapists, and advocacy groups to assist in home-based care and support. This professional team could coordinate in improving the autonomy and functional independence of people with ADRD, as well as the adaptation of the care environment, and could add great value to home-based care interventions.

Even though the government initiatives in many countries put restrictions in nursing home care facilities (e.g., banning visitors and limiting nonessential group activities) [54], specific guidance for home-based care settings for people with ADRD have yet to be developed systematically. Therefore, based on the identified challenges during COVID-19, we have proposed a list of recommendations related to pandemic situations (Figure 1) specifically catering to people with ADRD that calls for immediate action.

### 4.1. Structural Level

People diagnosed with ADRD aged 65 years and over may typically suffer from several underlying health conditions such as chronic respiratory difficulty, cardiovascular disease, diabetes, and hypertension compared to people of a similar age without dementia [58]. These diseases could increase the risk of infection by COVID-19. Thus, people with ADRD may continue staying at home depending on their circumstances and obtain suggestions from doctors remotely for any general health issues instead of frequent visits to the hospital [44].

In addition, taking into account the higher prevalence of respiratory and cardiovascular difficulties in this population group (and even more if they develop COVID-19), one of the key interventions would be training in energy-saving techniques, such as remote patient monitoring (RPM), that reduce fatigue during the performance of daily living activities (ADLs) for both patients and caregivers [58].

People with ADRD, who are either suspected or infected by COVID-19, can potentially benefit from an implementation of alternative care management strategies such as the Hospital-at-Home (HaH) model [59]. This service is a healthcare modality that administers specialized medical care to patients within their own homes. In the management of acute medical conditions that usually require hospitalization, HaH could be an efficient alternative that enables better alignment of health needs with higher patient satisfaction and at a lower cost [60].

The information related to viral infection has strongly influenced individuals’ behavioral responses among the older adults [2]. It is important to disseminate more positive news, as well as the story of recovered COVID-19 patients, especially with examples from people with ADRD [61]. For example, the COVID-19 SOS Alert, which is developed by the World Health Organization (WHO) to convey first-hand positive information or recovery news to the public, can be motivational and reduce psychosocial distress and panic [61,62].

Since family caregivers provide constant care and crucial daily support for members with ADRD, they might have been disproportionately affected by COVID-19. This scenario is rather common to those living in a rural setting where community resources are limited [63]. For example, people with ADRD and their caregivers residing in rural areas may experience difficulty responding to technology-based resources. Thus, the accessibility to a variety of communal ADRD home-based care resources should be disseminated to them to a greater extent.

As COVID-19 disrupted both formal and informal caregiving process, there is a need to establish regular and frequent contacts between family caregivers, home care providers, and personal support workers (trained and specialized in home care) [64] in order to obtain instructions easily while encountering any severe cases in people with ADRD. In extremely serious situations, the home-based care providers should be aware about the “catchment area” if the people with ADRD need to move to a different apartment, building, or even nearby locality for immediate treatment [65].

It is currently a critical time for governments at all levels to invest in resources that will support developing a robust and long-term service guideline for family caregivers [66]. The guideline can be developed by assessing previous formal and informal care resources and determining whether they meet current needs, because care needs often change over time with the progression of the disease [65,67]. As people with ADRD affected by COVID-19 will continue to rely mostly on their caregivers, there is a need to consider supporting the workforce to provide community-based services.

### 4.2. Personal Level

People with ADRD often require support for ADLs and their need for support can be further propelled by stress and anxiety related to COVID-19 [44,57,67]. During a crisis, such as during a pandemic, the knowledge and skill of formal and informal caregivers associated with ADLs are important to quality of care. Primary caregiver nurses are in an excellent position in such circumstances to provide training on care management to empower informal caregivers [68]. By implementing that knowledge, informal caregivers can ensure both a comfortable involvement of people with ADRD in daily activities as well as manage their behavioral changes.

The multifaceted care needs of people with ADRD in the emergency phase of a pandemic become even more challenging. Some easy-to-follow written reminders along with relevant symbolic instructions on personal hygiene practices (e.g., hand washing procedure) or information on preventive measures (e.g., using face masks) can be helpful [30,44,57,67]. In addition, family caregivers can produce a do-it-yourself card set or post-it notes with simple instructions for daily activities to give to people with ADRD. It is recommended that these supportive materials should be placed at frequently visited areas at home (e.g., on the fridge or on the door), so that the patients can get easy access to them.

As discussed in above section, social isolation may disproportionately affect people with ADRD who do not have close family members or friends and rely on the support of voluntary services or social care, such as daycare venues, community centers, and places of worship [31,35]. This implies that the isolation process can disrupt meaningful personal relationships and result in a greater risk of loneliness for people with ADRD [44]. Caregivers can initiate participatory and recreation activities for people with ADRD such as physical activity, gamification, virtual meetings, or digital storytelling [22,69]. In addition, different digital resources such as telephone calls and online-based cognitive behavioral therapies could be harnessed in order to protect their social networks and enhance a sense of belonging [70]. Furthermore, it is important to develop advanced care plans considering the wishes and unique needs of people with ADRD to utilize readily available resources [44,64]. Finally, family caregivers should share and exchange their experiences with friends and neighbors who are also taking care of people with ADRD at home to promote good practices for home-based care.

In contrast, the positive utilization of social isolation can also be useful in developing quality interactions between family members and the person with ADRD that can strengthen inter-personal solidarity and subsequently alleviate inter-generational gaps [69]. Before the pandemic, some family members may have been busy with other commitments, but now, they have the opportunity to enjoy a higher degree of freedom to build a stronger relationship with their family member with ADRD [31]. Thus, utilizing the benefit of the pandemic by spending more time together would help defend against overall mental distress.

For medical assistance, both conventional and new technologies should be prioritized to connect with people with ADRD and their caregivers [10,52,71]. Telehealth or eHealth services can be a useful tool, where applicable and available, in order to provide distance instructions, monitoring, surveillance, and follow-up treatments. Moreover, assistive devices such as movement sensors, fall detection, medication reminder alarms, visualizations of hygiene instructions on television, and voice recorded instructions can be used to maintain their independence as well as reduce worry for family caregivers [72,73,74]. The family caregivers should keep in contact with the nearest emergency care service providers to maintain regular updates of the conditions of people with ADRD [75]. At the same time, they should be in touch with the family doctor if the people with ADRD develop any symptoms of COVID-19 to get instructions regarding medication, hygiene, and personal cleanliness [67,72]. It might also be beneficial to have a video conversation between the doctor, caregivers, and the patient to discuss their experiences navigating through the pandemic while managing medical conditions, the encountered stress, and effective communication techniques between all parties to provide the best medical assistance to people with ADRD [31,72,76].

## 5. Conclusions

COVID-19 imposed restrictions on social engagement and changed the way we used to do our daily activities. This impact could promote increasing further care needs and challenges for people with moderate to severe ADRD. We anticipate that the care needs and challenges will not be the same in every local circumstance as it depends on associated death rates, available resources, societal context, and corresponding governmental responses. Another issue that needs to be considered is that ADRD is heterogeneous, which is the reason that any simple and generic recommendation for this group of people might not be effective. However, awareness of possible impacts and knowing details about the vulnerable risk group may prevent or reduce the harm of this pandemic on people with ADRD and their potential caregivers.

The governments of many countries have been trying to reduce the impact of pandemics and situations such as COVID-19, which will be under control with time despite major efforts required from everyone. While this uncertain pandemic situation has resulted in distressful experiences in the general population, we want to emphasize that these negative and anxious experiences will be magnified in people with ADRD, given their original medical conditions. Therefore, it gives us a realistic point to bring across ADRD home-based care as one of the topmost global priorities. We suggest a high priority need to strictly follow the guidelines recommended by Alzheimer’s Disease International and CDC [6,18], along with our recommendations to alleviate social and economic impact in times of pandemic crises.

## Abbreviations

ADRD: Alzheimer’s Disease and Related Dementia; CDC: Centers for Disease Control and Prevention; COVID-19: Coronavirus Disease 2019; HaH: Hospital-at-Home; RPM: Remote Patient Monitoring; SARS: Severe Acute Respiratory Syndrome.

## References

[1] Li Q., Guan X., Wu P., Wang X., Zhou L., Tong Y., Ren R., Leung K.S., Lau E.H., Wong J.Y. (2020). Early transmission dynamics in Wuhan, China, of novel coronavirus–infected pneumonia. N. Engl. J. Med..

[2] Onder G., Rezza G., Brusaferro S. (2020). Case-fatality rate and characteristics of patients dying in relation to COVID-19 in Italy. JAMA.

[3] Donnelly S. (2020). The elderly and COVID-19: Cocooning or culling—the choice is ours. QJM Int. J. Med..

[4] Alzheimer’s Disease International (ADI) (2019). World Alzheimer Report 2019: Attitudes to Dementia. Alzheimer’s Disease Internationals.

[5] Alzheimer’s Disease International (ADI) (2020). COVID-19 and Dementia: Difficult Decisions about Hospital Admission and Triage. https://www.alz.co.uk/sites/default/files/pdfs/ADI-position-paper-COVID-19-and-dementia.pdf.

[6] Alzheimer’s Disease International (ADI) (2020). Age, dementia and the allocation of health resources during and beyond COVID-19. https://www.alzint.org/u/Age-dementia-and-the-allocation-of-health-resources-during-and-beyond-COVID-19.pdf.

[7] Grasselli G., Pesenti A., Cecconi M. (2020). Critical care utilization for the COVID-19 outbreak in Lombardy, Italy: Early experience and forecast during an emergency response. JAMA.

[8] Jahan Y., Rahman A. (2020). COVID-19: Challenges and viewpoints from low-and-middle-income Asian countries perspectives. J. Saf. Sci. Resil..

[9] Lloyd-Sherlock P.G., Kalache A., McKee M., Derbyshire J., Geffen L., Casas F.G.-O., Gutierrez L.M. (2020). WHO must prioritise the needs of older people in its response to the covid-19 pandemic. BMJ.

[10] United Nations (2020). Policy Brief. The Impact of COVID-19 on Older Persons. https://unsdg.un.org/sites/default/files/2020-05/Policy-Brief-The-Impact-of-COVID-19-on-Older-Persons.pdf.

[11] McMichael T.M., Currie D.W., Clark S., Pogosjans S., Kay M., Schwartz N.G., Lewis J., Baer A., Kawakami V., Lukoff M.D. (2020). Epidemiology of Covid-19 in a long-term care facility in King County, Washington. N. Engl. J. Med..

[12] Livingston G., Sommerlad A., Orgeta V., Costafreda S.G., Huntley J., Ames D., Ballard C., Banerjee S., Burns A., Cohen-Mansfield J. (2017). Dementia prevention, intervention, and care. Lancet.

[13] What Is Alzheimer’s Disease?. https://www.psychiatry.org/patients-families/alzheimers/what-is-alzheimers-disease.

[14] Moya-Alvarado G., Gershoni-Emek N., Perlson E., Bronfman F.C. (2016). Neurodegeneration and Alzheimer’s disease (AD). What can proteomics tell us about the Alzheimer’s brain?. Mol. Cell. Proteom..

[15] Mubangizi V., Maling S., Obua C., Tsai A.C. (2020). Prevalence and correlates of Alzheimer’s disease and related dementias in rural Uganda: Cross-sectional, population-based study. BMC Geriatr..

[16] Normal Ageing vs Dementia. https://www.alzheimers.org.uk/about-dementia/symptoms-and-diagnosis/how-dementia-progresses/normal-ageing-vs-dementia.

[17] Demetia. https://www.who.int/news-room/fact-sheets/detail/dementia.

[18] People Who Are at Higher Risk for Severe Illness. https://www.cdc.gov/coronavirus/2019-ncov/need-extra-precautions/people-at-higher-risk.html.

[19] Ehni H.-J., Wahl H.-W. (2020). Six Propositions against Ageism in the COVID-19 Pandemic. J. Aging Soc. Policy.

[20] Bauer K., Schwarzkopf L., Graessel E., Holle R. (2014). A claims data-based comparison of comorbidity in individuals with and without dementia. BMC Geriatr..

[21] Zhou F., Yu T., Du R., Fan G., Liu Y., Liu Z., Xiang J., Wang Y., Song B., Gu X. (2020). Clinical course and risk factors for mortality of adult inpatients with COVID-19 in Wuhan, China: A retrospective cohort study. Lancet.

[22] Rahman A., Jahan Y. (2020). Defining a ‘Risk Group’and Ageism in the Era of COVID-19. J. Loss Trauma.

[23] Jahan Y., Rahman S., Rahman A. (2020). COVID-19: A case report from Bangladesh perspective. Respir. Med. Case Rep..

[24] Kotfis K., Williams Roberson S., Wilson J.E., Dabrowski W., Pun B.T., Ely E.W. (2020). COVID-19: ICU delirium management during SARS-CoV-2 pandemic. Crit. Care.

[25] Zheng Y.-Y., Ma Y.-T., Zhang J.-Y., Xie X. (2020). COVID-19 and the cardiovascular system. Nat. Rev. Cardiol..

[26] Avula A., Gill A., Nassar Re Nalleballe K., Siddamreddy S., Chalhoub M. (2020). Locked-In with COVID-19. J. Clin. Neurosci..

[27] Coleman J.J., Manavi K., Marson E.J., Botkai A.H., Sapey E. (2020). COVID-19: To be or not to be; that is the diagnostic question. Postgrad. Med. J..

[28] Azarpazhooh M.R., Amiri A., Morovatdar N., Steinwender S., Ardani A.R., Yassi N., Biller J., Stranges S., Tokazebani M., Neya S.K. (2020). Correlations between COVID-19 and burden of dementia: An ecological study and review of literature. J. Neurol. Sci..

[29] O’Shea E. (2020). Remembering people with dementia during the COVID-19 crisis. HRB Open Res..

[30] Canevelli M., Valletta M., Blasi M.T., Remoli G., Sarti G., Nuti F., Sciancalepore F., Ruberti E., Cesari M., Bruno G. (2020). Facing Dementia During the COVID-19 Outbreak. J. Am. Geriatr. Soc..

[31] Hwang T.-J., Rabheru K., Peisah C., Reichman W., Ikeda M. (2020). Loneliness and Social Isolation during the COVID-19 Pandemic. Int. Psychogeriatr..

[32] Bocskor A., Hunyadi M., Vince D. (2017). National Academies of Sciences, Engineering, and Medicine (2015) The Integration of Immigrants into American Society; The National Academies Press: Washington, DC, USA, 458p. Intersect. East Eur. J. Soc. Politics.

[33] Vahia I.V., Blazer D.G., Smith G.S., Karp J.F., Steffens D.C., Forester B.P., Tampi R., Agronin M., Jeste D.V., Reynolds C.F. (2020). COVID-19, mental health and aging: A need for new knowledge to bridge science and service. Am. J. Geriatr. Psychiatry.

[34] Yip P.S., Cheung Y., Chau P.H., Law Y. (2010). The impact of epidemic outbreak: The case of severe acute respiratory syndrome (SARS) and suicide among older adults in Hong Kong. Crisis J. Crisis Interv. Suicide Prev..

[35] Armitage R., Nellums L.B. (2020). COVID-19 and the consequences of isolating the elderly. Lancet Public Health.

[36] Greenberg N.E., Wallick A., Brown L.M. (2020). Impact of COVID-19 pandemic restrictions on community-dwelling caregivers and persons with dementia. Psychol. Trauma Theory Res. Pract. Policy.

[37] Rais N.C., Au L., Tan M. (2020). COVID-19 Impact in Community Care–A Perspective on Older Persons with Dementia in Singapore. J. Am. Med Dir. Assoc..

[38] World Health Organization (WHO) (2020). Preventing and Managing COVID-19 across Long-Term Care Services: Policy Brief.

[39] Jennings L.A., Laffan A.M., Schlissel A.C., Colligan E., Tan Z., Wenger N.S., Reuben D.B. (2019). Health care utilization and cost outcomes of a comprehensive dementia care program for Medicare beneficiaries. JAMA Intern. Med..

[40] Guberman N., Lavoie J.-P., Pepin J., Lauzon S., Montejo M.-E. (2006). Formal service practitioners’ views of family caregivers’ responsibilities and difficulties. Can. J. Aging/La Rev. Can. Du Vieil..

[41] Pierce M. The impact of COVID-19 on People who Use and Provide Long-Term Care in Ireland and Mitigating Measures. https://ltccovid.org/2020/04/15/the-impact-of-covid-19-on-people-who-use-and-provide-long-term-care-in-ireland-and-mitigating-measures/.

[42] Lamont R.A., Quinn C., Nelis S.M., Martyr A., Rusted J.M., Hindle J.V., Longdon B., Clare L. (2019). IDEAL Study Team: Self-esteem, self-efficacy, and optimism as psychological resources among caregivers of people with dementia: Findings from the IDEAL study. Int. Psychogeriatr..

[43] Hajek A., Brettschneider C., Lange C., Posselt T., Wiese B., Steinmann S., Weyerer S., Werle J., Pentzek M., Fuchs A. (2015). Longitudinal predictors of institutionalization in old age. PLoS ONE.

[44] Brown E.E., Kumar S., Rajji T.K., Pollock B.G., Mulsant B.H. (2020). Anticipating and Mitigating the Impact of COVID-19 Pandemic on Alzheimer’s Disease and Related Dementias. Am. J. Geriatr. Psychiatry.

[45] Alzheimer’s Disease International (ADI) (2015). Global Estimates of Informal Care. http://www.silviahemmet.se/wp-content/uploads/2018/07/Global-Estimates-Web-copy.pdf.

[46] Winblad B., Amouyel P., Andrieu S., Ballard C., Brayne C., Brodaty H., Cedazo-Minguez A., Dubois B., Edvardsson D., Feldman H. (2016). Defeating Alzheimer’s disease and other dementias: A priority for European science and society. Lancet Neurol..

[47] Miranda-Castillo C., Woods B., Orrell M. (2010). People with dementia living alone: What are their needs and what kind of support are they receiving?. Int. Psychogeriatr..

[48] Livingston E., Bucher K. (2020). Coronavirus disease 2019 (COVID-19) in Italy. JAMA.

[49] Ranney M.L., Griffeth V., Jha A.K. (2020). Critical supply shortages—the need for ventilators and personal protective equipment during the Covid-19 pandemic. N. Engl. J. Med..

[50] Wu J.T., Leung K., Bushman M., Kishore N., Niehus R., de Salazar P.M., Cowling B.J., Lipsitch M., Leung G.M. (2020). Estimating clinical severity of COVID-19 from the transmission dynamics in Wuhan, China. Nat. Med..

[51] Physical Activity and Older Adults. https://www.who.int/dietphysicalactivity/factsheet_olderadults/en/.

[52] Melander C., Olsson M., Jayousi S., Martinelli A., Mucchi L. (2019). Digital Resources Aiding Opportunities for Affiliation and Practical Reasoning Among People with Dementia: A Scoping Review. EAI International Conference on Body Area Networks.

[53] Wolff J.L., Feder J., Schulz R. (2016). Supporting family caregivers of older Americans. N. Engl. J. Med..

[54] Wang H., Li T., Barbarino P., Gauthier S., Brodaty H., Molinuevo J.L., Xie H., Sun Y., Yu E., Tang Y. (2020). Dementia care during COVID-19. Lancet.

[55] Tarricone R., Tsouros A.D. (2008). Home Care in Europe: The Solid Facts.

[56] Klosek M., Hall J., St-Amant O., Ward-Griffin C., DeForge R., Forbes D., Oudshoorn A., McWilliam C. (2012). Dementia home care resources: How are we managing?. J. Aging Res..

[57] Llibre-Guerra J.J., Jiménez-Velázquez I.Z., Llibre-Rodriguez J.J., Acosta D. (2020). The impact of COVID–19 on Mental Health in the Hispanic Caribbean Region. Int. Psychogeriatr..

[58] Zaman S., MacIsaac A.I., Jennings G.L., Schlaich M., Inglis S.C., Arnold R., Chew D.P., Kumar S., Thomas L., Wahi S. (2020). Cardiovascular disease and COVID-19: Australian/New Zealand consensus statement. Med. J. Aust..

[59] Leff B. (2009). Defining and disseminating the hospital-at-home model. Can. Med Assoc. J..

[60] Conley J., O’Brien C.W., Leff B.A., Bolen S., Zulman D. (2016). Alternative strategies to inpatient hospitalization for acute medical conditions: A systematic review. JAMA Intern. Med..

[61] Huang C., Xu X., Cai Y., Ge Q., Zeng G., Li X., Zhang W., Ji C., Yang L. (2020). Mining the Characteristics of COVID-19 Patients in China: Analysis of Social Media Posts. J. Med. Internet Res..

[62] Zarocostas J. (2020). How to fight an infodemic. Lancet.

[63] Williamson H.J., McCarthy M.J., Garcia Y.E., Bacon R., Dunn D.J., Baldwin J.A. (2020). Addressing the Needs of Rural Caregivers of Individuals With Alzheimer’s Disease and Related Dementias During and Beyond Coronavirus Disease 2019 (COVID-19). Public Policy Aging Rep..

[64] Hinton L., Tran D., Nguyen T.-N., Ho J., Gitlin L. (2019). Interventions to support family caregivers of people living with dementia in high, middle and low-income countries in Asia: A scoping review. BMJ Glob. Health.

[65] Quiñones A.R., Mitchell S.L., Jackson J.D., Aranda M.P., Dilworth-Anderson P., McCarthy E.P., Hinton L. (2020). Achieving health equity in embedded pragmatic trials for people living with dementia and their family caregivers. J. Am. Geriatr. Soc..

[66] Super N. (2020). Three trends shaping the politics of aging in America. Public Policy Aging Rep..

[67] Livingston G., Rostamipour H., Gallagher P., Kalafatis C., Shastri A., Huzzey L., Liu K., Sommerlad A., Marston L. (2020). Prevalence, management, and outcomes of SARS-CoV-2 infections in older people and those with dementia in mental health wards in London, UK: A retrospective observational. Lancet Psychiatry.

[68] Smith M., Gerdner L.A., Hall G.R., Buckwalter K.C. (2004). History, development, and future of the progressively lowered stress threshold: A conceptual model for dementia care. J. Am. Geriatr. Soc..

[69] Ayalon L., Chasteen A., Diehl M., Levy B., Neupert S.D., Rothermund K., Tesch-Römer C., Wahl H.-W. (2020). Aging in times of the COVID-19 pandemic: Avoiding ageism and fostering intergenerational solidarity. J. Gerontol. Ser. B.

[70] Newman M.G., Zainal N.H. (2020). The value of maintaining social connections for mental health in older people. Lancet Public Health.

[71] Rochford-Brennan H. (2020). Timely Psychosocial Interventions in Dementia Care: Evidence-Based Practice.

[72] Manthorpe J., Moniz-Cook E. (2020). Timely Support for People with Dementia: New Agendas and Challenges.

[73] Moon S., Park K. (2020). The effect of digital reminiscence therapy on people with dementia: A pilot randomized controlled trial. BMC Geriatr..

[74] Narasimha S. (2020). Forming Impressions on Computer-Mediated Healthcare Peer-Support Systems for Informal Caregivers.

[75] Giebel C., Cannon J., Hanna K., Butchard S., Eley R., Gaughan A., Komuravelli A., Shenton J., Callaghan S., Tetlow H. (2020). Impact of COVID-19 related social support service closures on people with dementia and unpaid carers: A qualitative study. Aging Ment. Health.

[76] Le Couteur D.G., Anderson R.M., Newman A.B. (2020). COVID-19 through the lens of gerontology. J. Gerontol. Ser. A Boil. Sci. Med. Sci..

